# Supplementary material for: Chromothripsis during telomere crisis is independent of NHEJ, and consistent with a replicative origin
Source: Genome Res. 2019 May;29(5):737–49. doi: 10.1101/gr.240705.118 (PMC6499312; doi:10.1101/gr.240705.118)
Supplement: Supplemental Material [file supp_gr.240705.118_Supplemental_file_1.zip › contigs/annotated_contigs/DB39/contig.2.DB39_length_541_mean_cov_5.94454713494.docx]

**DB39_length_541_mean_cov_5.94454713494**

```
TCTCCTTGAAGAGGTCCTTCACATCATTTGTAAGTTGGATTCCTAGGTATTTTGTTCTCTTTGTAATAATTGTGAATGGGAATTCACTC
 >chr10:81350464-81350671 - E=6e-110
ATGATTTGCCTCTCTATTATTGACGTATAGGAATGCTTGTGATTTTTGCACATTGATTTTGTATCCTGAGACTTTGCTGAAGTTGCTCA

TCAGCTTAAGGAGATTTTGGGCTGAGAC|A|TACATACTTCTCATGATTGGGAAAGAAACACAAAGGCCATAGCAAACCTGTAAGCACA
                             >chr10:81346626-81346961 - E=5e-190
CGTTAAGCTGCTGCTCCCTTCGTTTCAGCTCAGGGTCACATGGCCACACCAAACAGCAAAGGGGCTGGAGTGTTTACTCCTCACACTCC

AGTGGGAGCCTTTGTGAAGTCCTATGGCAAAGGGCCTGAGATGTAATTTTACAACATGGAGGAAACAACCCAAACTACCATGGCCATAT

CTGCTAAGTTTATGTCTCTTACAAAGAACCAGTATTCTATTTTATTAGCTCTATAGTTATTTTTGTTTATTAATTTAATTATATAATTT

TCATTCATT
```
